# Lack of pulmonary involvement leads to a 3 years delay of diagnosis in a childhood sarcoidosis case with arthritis, ocular symptoms, and bilateral parotid swelling

**DOI:** 10.1002/ccr3.8982

**Published:** 2024-06-05

**Authors:** Mohammed Taib Fatih, Renaz Sabir Saleh, Truska Faraidun Majeed, Mohammed Khalid Mahmood

**Affiliations:** ^1^ Dentistry Department Komar University of Science and Technology Kurdistan Iraq; ^2^ College of Dentistry, Oral Diagnosis Department Sulaimani University Kurdistan Iraq; ^3^ Aix‐Marseille University, CNRS, EFS, ADES Marseille France

**Keywords:** children, parotid sarcoidosis, parotid swelling, pulmonary sarcoidosis, sarcoidosis

## Abstract

**Key Clinical Message:**

This paper presents a rare sarcoidosis case in a child of 12 years of age presented with arthritis, bilateral parotid enlargement and ocular, but unfortunately the diagnosis has been missed due to lack of pulmonary involvement.

**Abstract:**

Diagnosis of sarcoidosis is by exclusion, and sometimes, it can be challenging. This paper presents a rare sarcoidosis case in a child of 12 years of age presented with bilateral parotid enlargement. The signs of musculoskeletal and ocular involvement were present before the parotid enlargement, and the parotid swelling persisted for 3 years; but unfortunately the definite diagnosis has been missed by the previous healthcare professionals most probably due to the rarity of the situation, especially lack of pulmonary involvement. Therefore, cooperation between different healthcare specialties is important for an effective diagnosis and management. Despite its rarity, sarcoidosis should always be present in the list of differential diagnosis when encountering multisystem entities like arthritis, ocular symptoms and parotid swelling.

## 1 INTRODUCTION

Sarcoidosis which is a multi‐systemic granulomatous disease with the hallmark of non‐caseating granulomas in multiple organs has an unclear etiology. The condition typically affects the skin, joints, and eyes in addition to causing bilateral hilar lymphadenopathy and granuloma in the lungs.^1^ Lungs are affected in more than 90% of cases.^2^ Although the precise cause of sarcoidosis is uncertain, exposure to the environment and working environments has been linked to the disease. There is currently no research that shows a clear causal link.^3^

Sarcoidosis primarily affects adults, though it can occasionally afflict kids as well. Although the incidence and prevalence in early children are unclear, the estimate is 10–20/100,000 individuals. The majority of instances affect patients between the ages of 8 and 15. Up until maturity, the prevalence rises, with the 20–40 age range being the most vulnerable. There is no known sex predilection among children with sarcoidosis.^4^

There are two different kinds of the condition in children. The trinity of a rash, uveitis, and arthritis is indicative of early‐onset sarcoidosis, which manifests before the age of five but lacks obvious pulmonary involvement. Older children typically have late‐onset sarcoidosis, which manifests as a multisystem illness with hilar lymphadenopathy and pulmonary infiltrate.^5^

Sarcoidosis rarely affects the parotid glands, with a prevalence less than 6% of the patients. A decrease in salivary production may result from the most prevalent symptom of salivary gland sarcoidosis, which is a painless, chronic swelling of the parotid glands.^6^

Parotid swelling has several other differential diagnosis, such as immune conditions (Sjogren's syndrome), salivary gland neoplasms, viral conditions (mumps, HIV), sialadenosis due to malnutrition or alcoholism, sialadenitis due to infection or obstruction, lymphoma, diabetes, and medication side effects.^7^

Sarcoidosis causes granulomatous inflammation and the differential diagnosis for granulomatous disease includes infections such as tuberculosis, syphilis, and fungal infections, as well as Crohn's disease and foreign body granulomas. In addition, based on the location, extent and severity of the disease, signs and symptoms of sarcoidosis overlap with many other conditions especially auto‐immune, inflammatory and hypersensitivity‐related diseases.

## 2 CASE PRESENTATION

**FIGURE 1:**
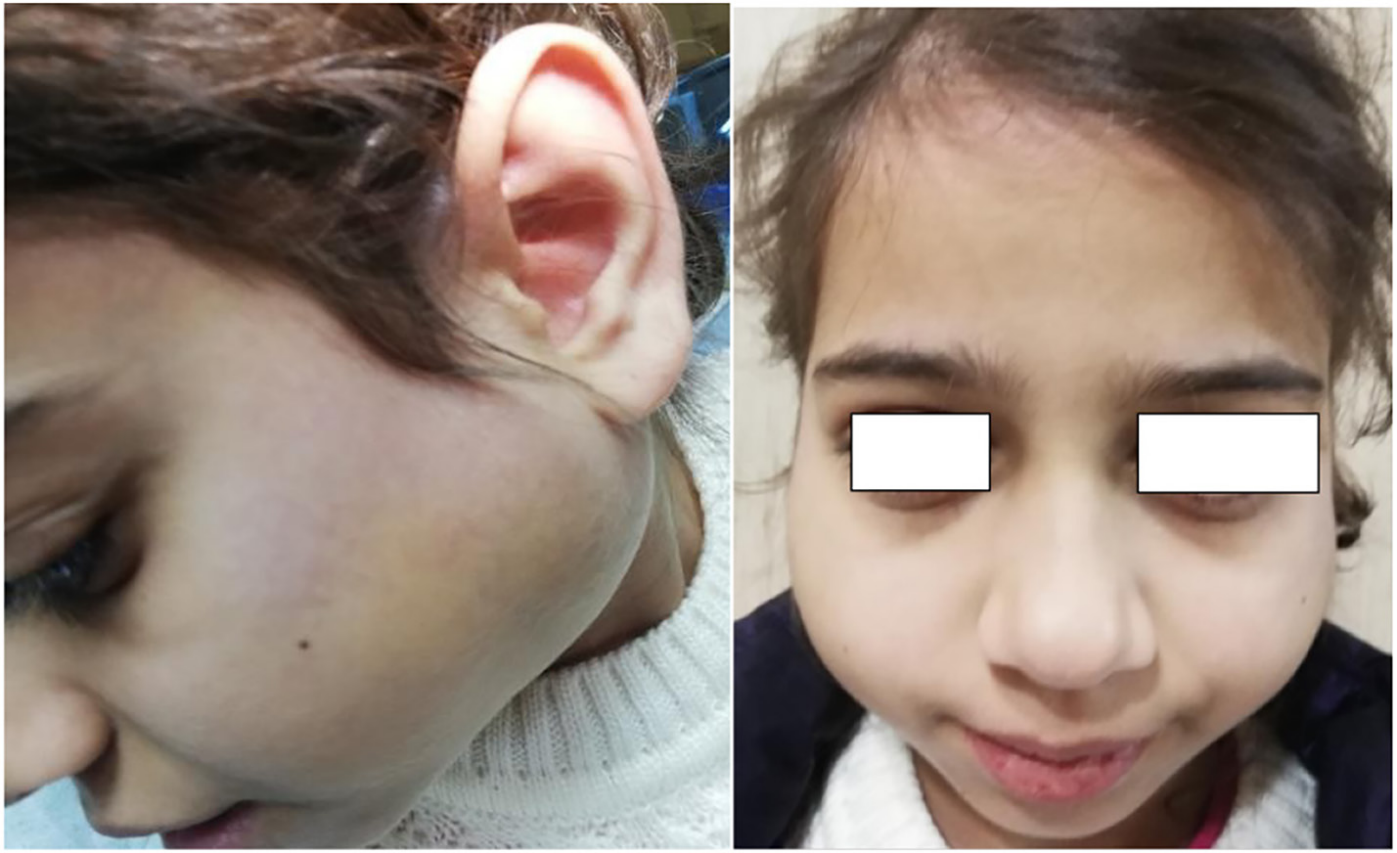
Lateral and frontal view of the swollen parotid and peri‐auricular area.

**FIGURE 2:**
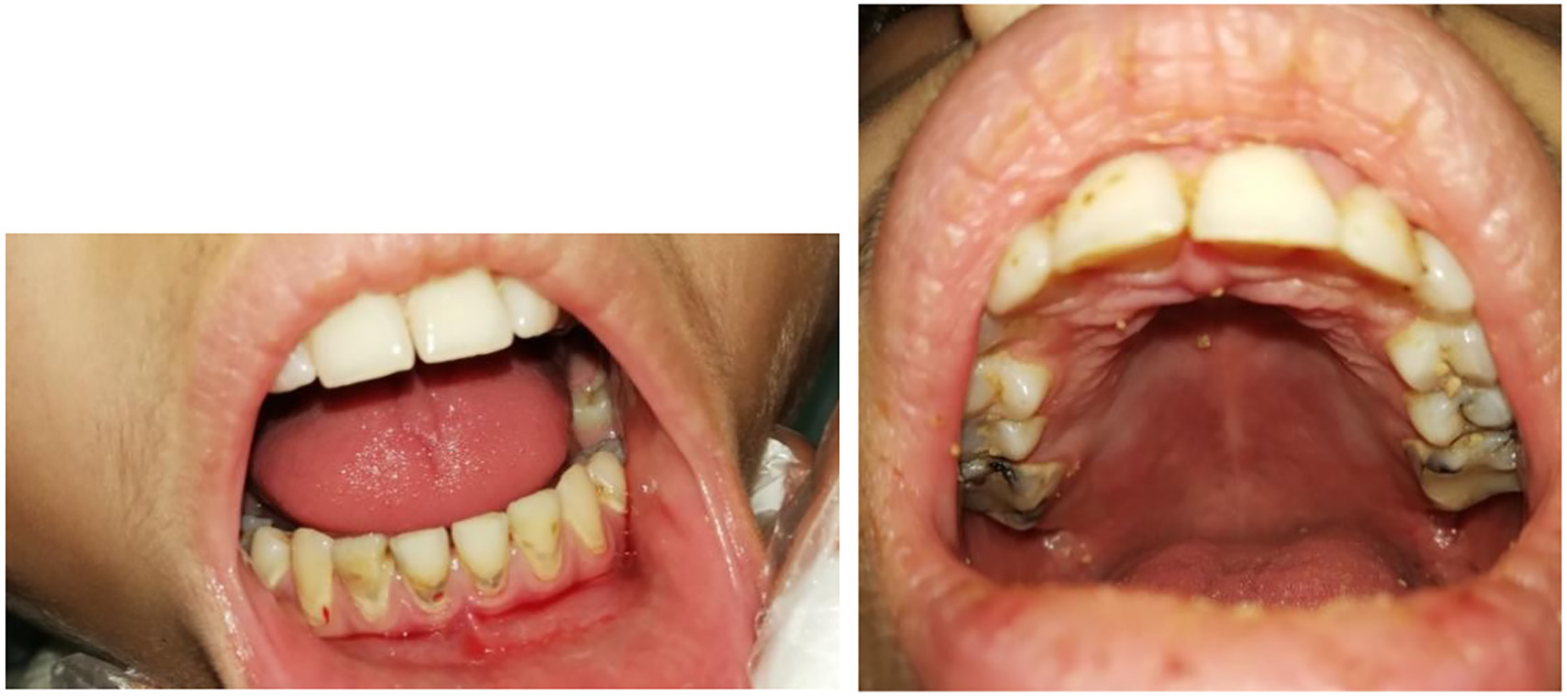
Intraoral view showing multiple cervical caries, large restorations over the permanent molars and remnants of food debris due to reduced washing action of saliva.

A 12‐year‐old girl presented to maxillofacial department of Shar hospital with painless bilateral slow‐growing posterior jaw swelling for 3 years. She had multiple consultation and treatment attempts without cure. Upon clinical examination, the symmetrically swollen parotid glands were firm, non‐movable, non‐pulsatile, non‐tender to palpation with normal colored, intact overlying facial skin. The other two major salivary glands were examined and were devoid of any palpable changes (Figure 1). Intraoral examination revealed signs of reduced salivary flow rate which included multiple cervical caries, large restorations over the permanent molars, remnants of food debris due to reduced washing action of saliva (Figure 2). Furthermore, her parents reported that she sips plenty of water with eating meals; if not, she is having difficulty in swallowing. Once dry mouth was suspected, chair‐side unstimulated salivary flow rate test was performed by spitting into a calibrated tube and the result was 0.2 mL/15 min.

Concerning the systemic symptoms her parents denied of noting any systemic upset like fever, malaise, cough, shortness of breath, loss of appetite, or weight loss, but the only complaint of the child was feeling of dryness in her eyes which necessitated visiting ophthalmologist whom prescribed eye drops to relieve symptoms.

Regarding the past medical history, about 4 years before (age 8) she once developed bilateral painful swelling of her knee joints, treated as emergency case by aspiration and painkillers.

## 3 METHODS

Based on the clinical examination, our list of the differential diagnosis included sarcoidosis, chronic bacterial sialadenitis, Crohn's disease, and other granulomatous infections like tuberculosis. First, chronic bacterial sialadenitis was excluded since the parotid swelling was persistent for 3 years despite several antibiotic treatments. Further investigations were performed to exclude the probabilities and reach a definite diagnosis.

Laboratory investigations which showed hypochromic microcytic anemia, raised serum glutamic oxaloacetic transaminase (GOT) and glutamic pyruvic transaminase (GPT), high erythrocyte sedimentation rate (ESR) level of 30 mm/h and negative C‐reactive protein (CRP). Autoimmune investigations were doubtful, but rheumatoid factors (IgG and IgM) were not positive.

Subsequently, ultrasonography of the neck showed enlarged parotid glands and multiple inflammatory cervical lymph node enlargements; however, no obvious abnormality was detected in the abdominal ultrasound. About 2 years later (age 10) her parents kept seeking for treatment of her facial swelling, recommended investigations were a neck ultrasound which showed markedly enlarged major salivary glands with diffuse micro calcifications which raised suspicion toward autoimmune sialadenitis.

**FIGURE 3:**
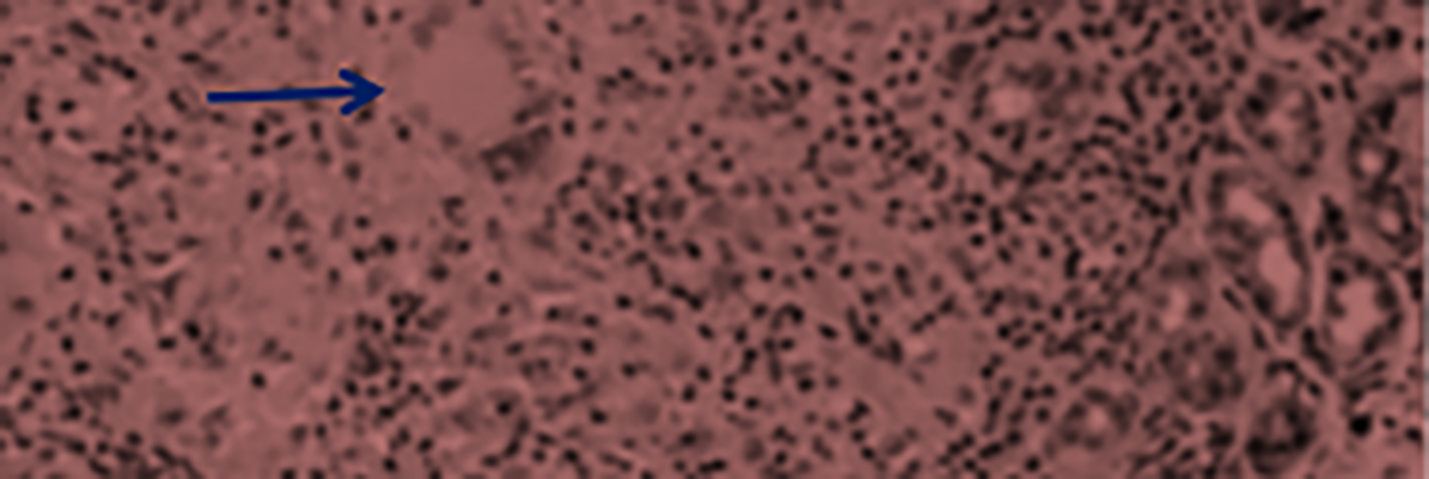
Image of labial salivary gland biopsy showing non‐caseating granuloma (the arrow) surrounded by a rim of lymphocytes.

In addition, fine‐needle aspiration cytology showed lymphoepithelial islands caused by infiltration of the ductal epithelial cells by lymphocytes with features consistent with autoimmune saialadenitis. Other serological investigations like creatinine, urea, ANA, C3, C5, and thyroid function tests were all within normal limit. Following those mentioned tests, incisional biopsy was carried out to reach the final diagnosis. The biopsy exhibited non‐caseating granulomas containing few Langhan's giant cells and sarcoid‐like granulomas (Figure 3). Finally, the chest x‐ray did not show any signs of hilar lymphadenopathy.

Sarcoidosis was the diagnosis by exclusion. The patient was treated by Prednisolone 20 mg qd.

## 4 OUTCOME AND FOLLOW‐UP

**FIGURE 4:**
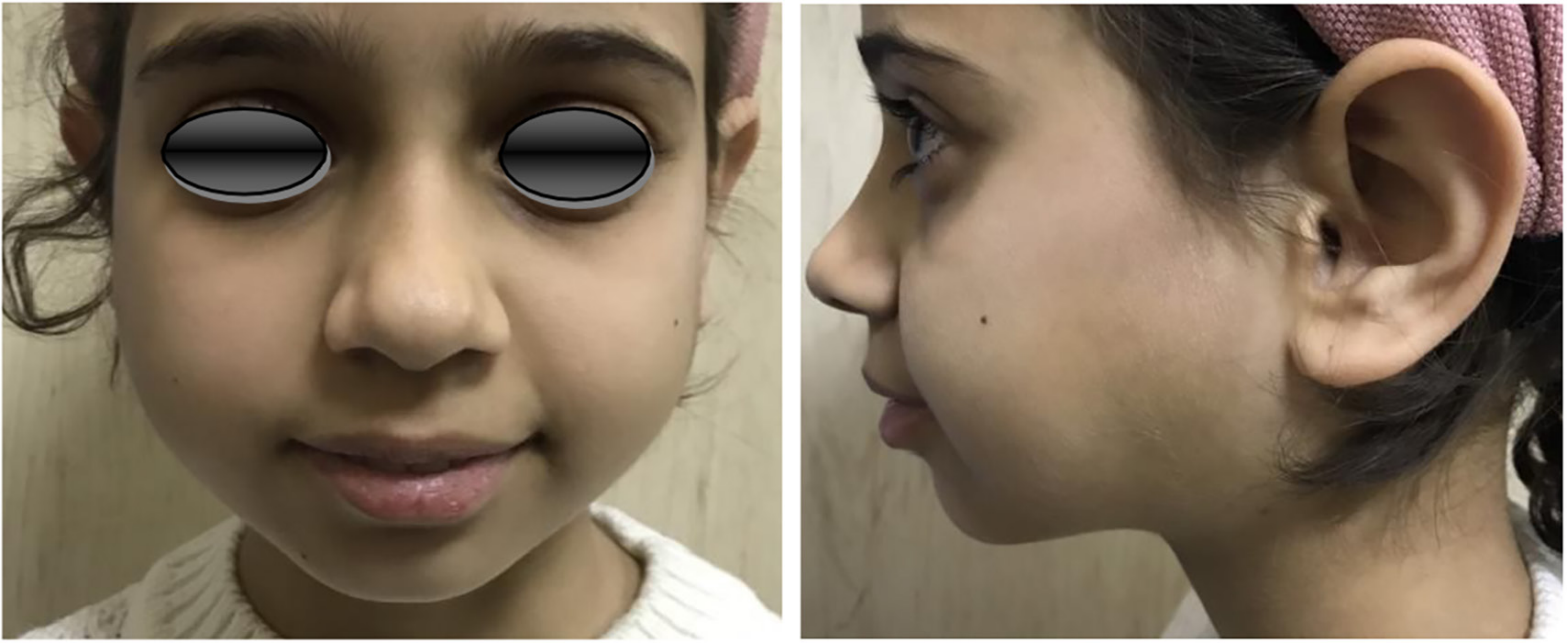
Follow‐up 10 days after treatment with Prednisolone showing significant reduction in the parotid swelling.

**FIGURE 5:**
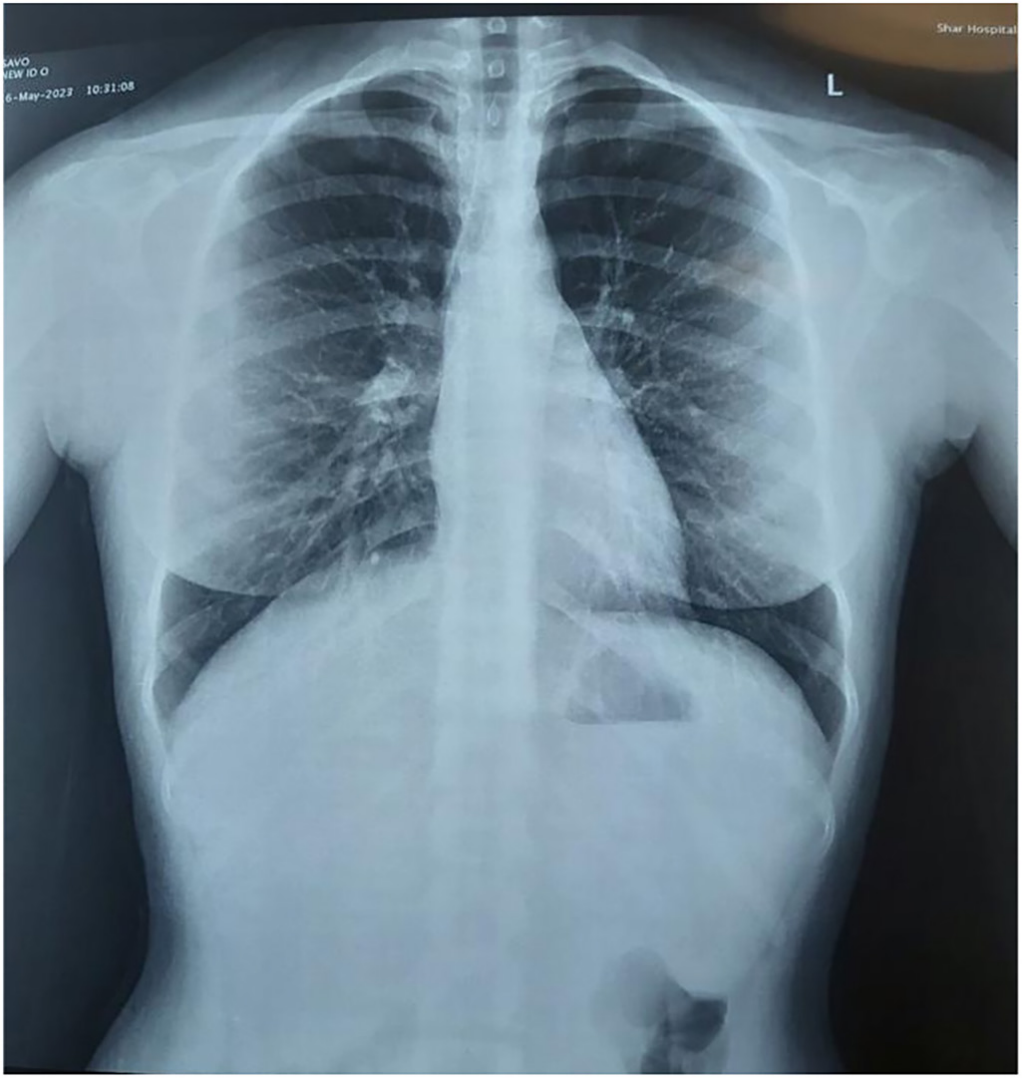
Chest x‐ray showing no signs of hilar lymphadenopathy.

Follow‐up after 10 days showed good response to treatment (Figure 4). She was regularly followed up. In the 2‐year follow‐up meeting, she was generally in a good health. Parotid swelling was absent. She had only a mild dryness sensation inside the mouth. On inspection, oral mucosa was relatively wet. The same was true for the eyes. She had no complains about her joints. A new chest x‐ray after 2 years still showed no signs of pulmonary involvement (Figure 5).

## 5 DISCUSSION

Sarcoidosis as a multi‐systemic systemic granulomatous condition with an unknown cause, primarily affects young adults and is comparatively uncommon in children. The frequency of clinically recognizable sarcoidosis in children was found to be 0.22 to 0.29/100,000 children annually in several large evaluations. The disease progressively increases with age, reaching a minor peak in teenagers between the ages of 13 and 15.^8^, ^9^

Children younger than 5‐year‐olds typically have the triad of skin, joint, and ocular involvement without the characteristic lung disease. However, older children, as observed in adults, more typically experience involvement of the eyes, lymph nodes, and lungs.^5^ Our case fits into the second category with the exception of lung involvement.

Lung is the mostly involved organ typically in the form of hilar lymphadenopathy and its prevalence is estimated to be over 90% of cases, although the clinical spectrum of the disease is wide.^10^ However chest x‐ray abnormalities were present only in 56% of 41 patients from France.^11^ In our case, the chest x‐ray was clear and pulmonary symptoms were absent. This may have contributed to the delay in reaching the diagnosis.

Arthritis is another component of sarcoidosis with different prevalence ranges. For example, joint manifestation in 52 Turkish pediatric patients was (25%).^12^ Moreover, pooled estimated prevalence of joint involvement in a total of 8574 patients was 19%.^13^ It appears that bilateral knee swelling and pain was the first sign of sarcoidosis in our case.

Prevalence of ocular sarcoidosis is reported as a range from 13 to 79%.^14^ Chronologically, the second sign of sarcoidosis in our case report was ocular involvement. Unfortunately, the patient was only treated with eye drops for rehydration. In addition, skin manifestations were present in approximately 25%–40% of pediatric cases.^5^ Our patient was devoid of cutaneous involvement. Furthermore, based on the absence of abdominal symptoms and the abdominal ultrasound, we excluded Crohn's disease. Renal function tests were normal, excluding the involvement of the kidneys.

Parotid gland involvement by sarcoidosis is extremely rare and is around 6%.^15^ In patients with parotid gland involvement, minor salivary gland biopsy can often be useful in making a diagnosis. Labial biopsy will show evidence of granulomatous changes in up to 58% of sarcoidosis patients with parotid involvement.^16^

There are several clinical patterns in which the involvement of the salivary glands can present. Major salivary gland swelling accompanied by minor salivary gland histologic involvement is the most prevalent pattern. There may be xerostomia, and its severity is closely correlated with how much granulomatous infiltration is present inside the gland.^10^, ^15^ The lack of clinical salivary gland swelling identifies a second pattern of salivary gland involvement. However, histologic analysis of small salivary gland biopsy specimens typically reveals the presence of non‐caseating granulomas regardless of the presence of swelling.^17^

Patients who have sarcoid infiltration of the major salivary glands and the xerostomia that follows are at risk for developing periodontal disease, candidiasis, and dental caries. Adequate oral hygiene is essential, including the use of strong preventative treatments including topical fluoride, salivary stimulants, and antifungal medications when needed.^17^ In our case, a remarkably reduced salivary flow accompanied with multiple cervical caries was noted.

Diagnosis of sarcoidosis is by exclusion and it can be challenging.^18^ Our case is a good example of a delay in diagnosis especially when the disease does not manifest in a typical pattern. Therefore cooperation between different healthcare specialties is important for an effective diagnosis and management. Despite its rarity, sarcoidosis should always be present in the list of differential diagnosis when encountering multisystem entities like arthritis, ocular symptoms, and parotid swelling.

In conclusion, in this paper, we reported a rare sarcoidosis case in a child of 12 years of age presented with bilateral parotid enlargement. The signs of musculoskeletal and ocular involvement were present before the parotid enlargement, and the parotid swelling persisted for 3 years; but unfortunately the definite diagnosis has been missed by the previous healthcare professionals most probably due to the rarity of the situation especially lack of pulmonary involvement.

## AUTHOR CONTRIBUTIONS

**Mohammed Taib Fatih:** Conceptualization; data curation. **Renaz Sabir Saleh:** Conceptualization; resources; validation. **Truska Faraidun Majeed:** Data curation; investigation; resources. **Mohammed Khalid Mahmood:** Investigation; methodology; writing – original draft; writing – review and editing.

## FUNDING INFORMATION

There is no funding or support for our article.

## CONFLICT OF INTEREST STATEMENT

There is no conflict of interest between authors.

## CONSENT

Written informed consent was obtained from the patient to publish this report in accordance with the journal's patient consent policy.

## Data Availability

Research data can be shared upon request.

## References

[1] Statement on sarcoidosis. Joint Statement of the American Thoracic Society (ATS), the European Respiratory Society (ERS) and the World Association of Sarcoidosis and Other Granulomatous Disorders (WASOG) adopted by the ATS Board of Directors and by the ERS Executive Committee, February 1999. Am J Respir Crit Care Med. 1999;160(2):736‐755. 10430755 10.1164/ajrccm.160.2.ats4-99

[2] Lynch JP III , Kazerooni EA , Gay SE . Pulmonary sarcoidosis. Clin Chest Med. 1997;18(4):755‐785. 9413657 10.1016/s0272-5231(05)70417-2

[3] Chen ES , Moller DR . Etiology of sarcoidosis. Clin Chest Med. 2008;29(3):365‐377. 18539232 10.1016/j.ccm.2008.03.011

[4] Arkema EV , Cozier YC . Epidemiology of sarcoidosis: current findings and future directions. Ther Adv Chronic Dis. 2018;9(11):227‐240. 30364496 10.1177/2040622318790197 PMC6196636

[5] Shetty AK , Gedalia A . Childhood sarcoidosis: a rare but fascinating disorder. Pediatr Rheumatol Online J. 2008;6:1‐10. 18811966 10.1186/1546-0096-6-16 PMC2559831

[6] Banks GC , Kirse DJ , Anthony E , Bergman S , Shetty AK . Bilateral parotitis as the initial presentation of childhood sarcoidosis. Am J Otolaryngol. 2013;34(2):142‐144. 23102965 10.1016/j.amjoto.2012.08.007

[7] Arnold DE , Heimall JR . A review of chronic granulomatous disease. Adv Ther. 2017;34:2543‐2557. 29168144 10.1007/s12325-017-0636-2 PMC5709447

[8] Gedalia A , Khan TA , Shetty AK , Dimitriades VR , Espinoza LR . Childhood sarcoidosis: Louisiana experience. Clin Rheumatol. 2016;35:1879‐1884. 25616361 10.1007/s10067-015-2870-9

[9] Milman N , Hoffmann A , Byg KE . Sarcoidosis in children. Epidemiology in Danes, clinical features, diagnosis, treatment and prognosis. Acta Paediatr. 1998;87(8):871‐878. 9736236 10.1080/080352598750013662

[10] Nathan N , Sileo C , Calender A , et al. Paediatric sarcoidosis. Paediatr Respir Rev. 2019;29:53‐59. 30917882 10.1016/j.prrv.2018.05.003

[11] Nathan N , Marcelo P , Houdouin V , et al. Lung sarcoidosis in children: update on disease expression and management. Thorax. 2015;70:537‐542. doi:10.1136/thoraxjnl-2015-206825 25855608

[12] Guliyeva V , Demirkan FG , Yiğit RE , et al. A clinical overview of paediatric sarcoidosis: multicentre experience from Turkey. Mod Rheumatol. 2023;34:639‐645. 10.1093/mr/road050 37243724

[13] Yeung T , Grebowicz A , Nevskaya T , Zahid S , Pope JE . Joint involvement in sarcoidosis: systematic review and meta‐analysis of prevalence, clinical pattern and outcome. Rheumatology. 2024 (Article in Press). 10.1093/rheumatology/keae048 38281070

[14] Pasadhika S , Rosenbaum JT . Ocular sarcoidosis. Clin Chest Med. 2015;36(4):669‐683. 26593141 10.1016/j.ccm.2015.08.009 PMC4662043

[15] James D , Sharma O . Parotid gland sarcoidosis. Sarcoidosis Vasc Diffuse Lung Dis. 2000;17(1):27‐32. 10746259

[16] Nessan VJ , Jacoway JR , Lauttman RJ , Swintak EF , Buhler JE Jr . Biopsy of minor salivary glands in the diagnosis of sarcoidosis. N Engl J Med. 1979;301(17):922‐924. 481539 10.1056/NEJM197910253011705

[17] Rao V , Curran J , Blair EA , Sweiss NJ . Salivary glands sarcoidosis. Oper Tech Otolaryngol Head Neck Surg. 2008;19(4):234‐236.

[18] Chiu B , Chan J , Das S , Alshamma Z , Sergi C . Pediatric sarcoidosis: a review with emphasis on early onset and high‐risk sarcoidosis and diagnostic challenges. Diagnostics. 2019;9(4):160. 31731423 10.3390/diagnostics9040160 PMC6963233

